# Effects of simvastatin pretreatment on platelet activation and hypercoagulable state in septic mice

**DOI:** 10.1186/s40001-025-02677-2

**Published:** 2025-05-27

**Authors:** Fan Wu, Ke Xu, Wei Xiong

**Affiliations:** 1 Department of Clinical Laboratory, Shanghai Mengchao Cancer Hospital, Shanghai, 201805 China; 2 Department of Clinical Laboratory, Shanghai Tenth People’s Hospital, 3rd Floor, Building 1, 301 Yanchang Middle Road, Jing’an District, Shanghai, 200072 China

**Keywords:** Simvastatin, Sepsis, Mouse, Platelet activation, Hypercoagulant state

## Abstract

**Objective:**

To investigate the effects of simvastatin pretreatment on platelet activation and hypercoagulable state in septic mice.

**Method:**

60 Sprague–Dawley (SD) mice were divided into three groups: healthy control (group A), sepsis (group B) and simvastatin intervention (group C). The sepsis model was established by intraperitoneal injection of lipopolysaccharide in the group B: the group A was injected with normal saline, and the group C was injected with 10 μg/ml simvastatin 5 ml of simvastatin for 3 h. The changes of protein expression were detected by Western blot, blood coagulation indexes were analyzed, and the levels of serum platelet activating factor, thrombomodulin, interleukin 6 (IL-6), tumor necrosis factor-α (TNF-α), interleukin-1 β (IL-1β), superoxide dismutase (SOD) and malondialdehyde (MDA) were detected by enzyme linked immunosorbent assay (ELISA) detection kit.

**Result:**

The levels of thrombomodulin and platelet activating factor in mice of the group B were significantly higher than those in the group A (*P* < 0.05). The levels of thrombomodulin and platelet activating factor in the group C of mice were significantly lower than those in the group B (*P* < 0.05), which were down-regulated by 20.44% and 33.33%, respectively (*P* < 0.05). The PT and APTT times of mice in the group B were significantly lower than those in the group A (*P* < 0.05). The PT and APTT times of mice in the group C were significantly higher than those in the group B (*P* < 0.05), which were upregulated by 29.01% and 13.08%, respectively (*P* < 0.05). The SOD level of mice in the group B was significantly lower than that in the group A, and the MDA level was significantly higher than that in the group A (*P* < 0.05). The SOD level in the group C of mice was significantly higher than that in the group B, which was upregulated by 24.77%, and the MDA level was significantly lower than that in the group B, which was down-regulated by 22.96% (*P* < 0.05). The levels of serum IL-6, TNF-α and IL-1β in mice of the group B were higher than those in the group A (*P* < 0.05). The levels of serum IL-6, TNF-α and IL-1β in the group C of mice were lower than those in the group B by 45.97%, 28.72% and 16.59%, respectively (*P* < 0.05). The expression levels of AMPK and UCP2 proteins in the group B of mice were lower than those in the group A (*P* < 0.05). The expression levels of AMPK and UCP2 proteins in the group C of mice were higher than those in the group B, which were upregulated by 55.00% and 28.81%, respectively (*P* < 0.05).

**Conclusion:**

Simvastatin pretreatment can improve platelet activation and hypercoagulable state in septic mice.

## Introduction

Sepsis is a systemic inflammatory response syndrome (SIRS) caused by infection and tissue injury, with high morbidity and mortality [1]. Sepsis has the advantages of rapid progress, high clinical mortality, and difficult clinical diagnosis and treatment if it cannot be detected in time [2]. Studies have found that patients with sepsis are prone to secondary compensatory anti-inflammatory response syndrome (CARS) during the regression of inflammatory storm, which leads to secondary infection dominated by immune paralysis, resulting in a poor prognosis [3]. Although inflammation is at the core of the pathogenesis of sepsis, many other biological systems contribute to the pathology of the disease [4]. Statins are 3-hydroxy-3-methylglutaryl- Certificate of Analysis (CoA) reductase inhibitors, which can reduce blood lipids and have multiple effects, including improving endothelial dysfunction, reducing inflammation and inhibiting platelet activation. Studies have shown that simvastatin has immunomodulatory and anti-inflammatory effects, which do not depend on lipid-lowering effects [5]. In addition, previous studies have focused on the effect of statins on single inflammatory or coagulation indicators, but evidence on whether they can simultaneously regulate the coupled response of "inflammation–oxidation–hypercoagulability" by activating the "AMPK/UCP2-energy sensing pathway" is still lacking [6–8]. This study is the first time to systematically quantify the chain changes in the above three links in a mouse model of sepsis and evaluate the early regulatory effect of simvastatin on relevant signal axes, aiming to provide a new basis for precise anticoagulation-anti-inflammatory treatment of sepsis. Experimental basis.

## Materials and methods

### Establishing an animal model

Randomization method: SPSS20.0 was used to generate a random number sequence from 1 to 60, and assigned to three groups according to the sequence size. The operator and the tester were double-blinded.

Sample size estimation: Based on the PAF effect size obtained from previous preliminary experiments (*d* = 0.8), calculated using PASS20.0 (*α* = 0.05, efficacy = 0.90), a minimum of 18 animals per group is required, and 20 animals per group are finally determined.

60 SD mice were randomly divided into 3 groups: healthy control group, sepsis group and simvastatin intervention group, 20 mice in each group. Rats were anesthetized by intraperitoneal injection of 30 mg/kg of 1% pentobarbital sodium and fixed on an operating board. ECG was connected and monitored. Sepsis group was injected with LPS intraperitoneally to prepare mini-sepsis model; healthy control group was injected with the same volume of normal saline intraperitoneally; simvastatin intervention group was injected with simvastatin 5 ml at 10 μg/ml, and LPS was injected intraperitoneally 3 h later to prepare mini-sepsis model.

### Sampling and sample preparation

The venous blood 5 mL of mice was taken under anesthesia, and then the abdominal aorta was removed by rapid bloodletting, washed repeatedly with normal saline, and transferred to the refrigerator at − 80 ℃ for examination.

### Blood coagulation index

2 mL was extracted from fasting peripheral venous blood of mice, and activated partial thromboplastin time (APTT) and prothrombin time (PT) were detected by automatic coagulation analyzer (ACLTOP700, Wolfen, USA).

### Detection of protein expression by western blot

At 4 °C, abdominal aorta tissue was homogenized to make a 10% homogenate, and the supernatant was centrifuged for analysis. We determined the protein concentration using the bicinchoninic acid assay (BCA) method, gel preparation, electrophoresis for 90 min, gel cutting, membrane transfer for 90 min, milk sealing, washing, and primary and secondary antibodies incubation.

### Serological index

20 min was separated by 3000 r/min in venous blood, and the supernatant was reserved for examination. The levels of factors were detected strictly according to the instructions of ELISA kit.

### Statistical method

Statistical analysis was carried out using SPSS20.0, and the measurement data were expressed as mean ± standard deviation ($\overline{x} \pm s$). One-way analysis of variance was first used to compare the three groups, and Levene's test was used to verify the homogeneity of variances; when homogeneity was met, LSD-t was used for pairwise comparisons, and Tamhane-T2 was used for non-homogeneity. In order to reduce the bias of multiple comparisons, the Benjamini–Hochberg method was used to correct the false discovery rate (FDR) for the *P* value of pairwise comparisons of the same indicator. The FDR ≤ 0.10 was judged as statistically significant, and the difference was statistically significant with *P* < 0.05.

## Result

### The levels of thrombomodulin and platelet activating factor

The levels of thrombomodulin and platelet activating factor in group B were raised than group A, and these in group C were reduced than group B (Table 1).

**Table 1:** Comparison of the levels of thrombomodulin and platelet activating factor

Group	*N*	Thrombomodulin (ng/mL)	Platelet activating factor (pg/mL)
A	20	0.9 ± 0.33	13.97 ± 3.83
B	20	1.81 ± 0.63^a^	27.51 ± 2.13^a^
C	20	1.44 ± 0.45^b^	18.34 ± 1.25^b^
F		25.587	1244.373
P		0.000	0.000

Compared with healthy control group: ^a^*P* < 0.05; compared with sepsis group: ^b^*P* < 0.05 (*P* value is still significant after correction by Benjamini–Hochberg method)

### Comparison of coagulation indexes in mice

The PT and APTT time in group B were decreased than group A, while these in group C were increased than group B (Table 2).

**Table 2:** Comparison of coagulation indexes in mice

Group	*N*	PT(s)	APTT(s)
A	20	13.37 ± 4.09	33.54 ± 4.03
B	20	8.86 ± 2.16^a^	24.31 ± 3.03^a^
C	20	11.43 ± 3.14^b^	27.49 ± 4.24^b^
F		80.413	158.235
P		0.000	0.000

Compared with healthy control group: ^a^*P* < 0.05; compared with sepsis group: ^b^*P* < 0.05 (*P* value is still significant after correction by Benjamini–Hochberg method)

### Comparison of oxidative stress indexes in mice

The SOD in group B was reduced group A, and the MDA was raised than group A. The SOD in group C was increased than group B, and the MDA in group C was decreased than group B (Table 3).

**Table 3:** Oxidative stress indexes

Group	*N*	SOD (kU/g)	MDA (μmol/g)
A	20	88.38 ± 14.32	4.14 ± 1.13
B	20	51.52 ± 12.49^a^	8.23 ± 2.69^a^
C	20	64.28 ± 14.39^b^	6.34 ± 1.47^b^
F		37.033	23.553
P		0.000	0.000

Compared with healthy control group: ^a^*P* < 0.05; compared with sepsis group: ^b^*P* < 0.05 (*P* value is still significant after correction by Benjamini–Hochberg method)

### Comparison of serum inflammatory indexes in mice

The serum levels of TNF-α, IL-6, and IL-1β in the group B were raised than group A, while these in the group C were decreased than group B (Table 4).

**Table 4:** Serum inflammatory indexes in mice

Group	*N*	IL-6 (μg·^L−1^)	TNF-α (ng·^L−1^)	IL-1β (pg·^L−1^)
A	20	97.86 ± 11.28	50.71 ± 8.22	9.34 ± 1.76
B	20	180.28 ± 18.04^a^	170.33 ± 17.11^a^	30.28 ± 4.84^a^
C	20	150.37 ± 16.09^b^	121.41 ± 13.14^b^	16.36 ± 2.13^b^
F		146.788	407.156	219.423
P		0.000	0.000	0.000

Compared with healthy control group: ^a^*P* < 0.05; compared with sepsis group: ^b^*P* < 0.05 (*P* value is still significant after correction by Benjamini–Hochberg method)

### Comparison of adenosine 5‘-monophosphate (AMP)-activated protein kinase (AMPK) and uncoupling protein 2 (UCP2) protein in mice

The AMPK and UCP2 protein in group B were decreased than group A, while these in group C were higher than group B (Table 5).

**Table 5:** Comparison of AMPK and UCP2 protein in mice

Group	*N*	AMPK	UCP2
A	20	0.68 ± 0.20	0.83 ± 0.35
B	20	0.40 ± 0.18^a^	0.59 ± 0.17^a^
C	20	0.62 ± 0.24^b^	0.76 ± 0.23^b^
F		10.031	4.474
P		0.000	0.016

Compared with healthy control group: ^a^*P* < 0.05; compared with sepsis group: ^b^*P* < 0.05 (*P* value is still significant after correction by Benjamini–Hochberg method)

## Discussion

Sepsis is a systemic inflammatory syndrome in which the inflammatory response to infection can lead to multiple organ dysfunction [9]. The liver is one of the target organs of sepsis, more than 1/3 of patients with sepsis are prone to liver dysfunction; the precursor is progressive hyperbilirubinemia. The study found that liver failure occurred in up to 22% of patients with sepsis, which confirmed the prognostic role of the liver in this septic condition [10]. Additionally, coagulation disorders are always associated with severe infection and are responsible for organ failure, which can lead to sepsis-related death. However, the findings of the study suggest that the positive effect of anticoagulants on organ microthrombosis is offset by the inherently high risk of bleeding associated with the treatment [11].

As a severe negative prognostic outcome of severe sepsis, progressive thrombocytopenia and coagulation disorders have recently been included in the latest definition. A destructive coagulation syndrome, diffuse intravascular coagulation, and systemic microvascular thrombosis are the extreme phenotypes of pathological coagulation in sepsis. By understanding the mechanisms of sepsis can be better understood [12]. Sepsis can be treated by addressing vascular endothelial dysfunction [13]. In animal models of liver causing vascular endothelial dysfunction. Nitric oxide vasodilation dependent on hepatic sinusoids is defined as vascular endothelial dysfunction. These studies, which mainly focus on endothelial vasomotor function, have entered phase II randomized controlled trials [14]. Importantly, simvastatin can improve the vasomotor function of sinus endothelial cells, which is one of the markers of the preservation of endothelial antithrombotic function in microcirculation. As a result, some scholars believe simvastatin has anticoagulant properties that can protect the liver from microthrombosis [15].

Anticoagulants provide survival benefits in highly selective patients, but at the expense of major bleeding complications in phase II and phase III trials [16]. The antithrombotic effect is achieved by simvastatin, which retains the endothelial expression of TM. Several clinical benefits can be derived from this mechanism [17]. Initially, the blood thinning encouraged by this technique is natural, as it does not specifically aim at coagulation components, therefore it will not disrupt the precise hemostatic feedback control mechanism. In conclusion, this anticoagulation strategy has a relatively low bleeding risk, although further studies on bleeding models will be required to verify this [18]. The results of this study found that the levels of thrombomodulin, platelet activating factor, MDA and IL-6, TNF-α, and IL-1β in mice in the group C were lower than those in the group B. PT, APTT and SOD in mice treated with simvastatin were significantly higher than those in group B. It is suggested that simvastatin pretreatment can improve inflammatory reaction, platelet activation and hypercoagulable state in septic mice. The multiple effects of statins are mainly based on the inhibition of isoprenoid synthesis, which is a biochemical pathway that regulates post-translational factors through isoprene [19]. Interference with this cellular control system by statins leads to the upregulation/downregulation of several enzymes [17].

There are various metabolic organs and tissues that express AMPK, which belongs to the serine/threonine protein kinase family. A variety of stimuli by sensing changes in cell energy metabolism, thereby affecting a number of links in the body's metabolism and energy balance. It can be activated by [20]. When metabolism and energy are imbalanced, AMPK activation can regulate downstream malonyl-CoA and lipid synthesis genes through phosphorylation, inhibiting fatty acid biosynthesis [21]. Additionally, AMPK and UCP2 proteins have been shown to be functionally correlated. The activity of mitochondrial enzymes can be enhanced as well as the efficiency of oxidative phosphorylation, the expression of UCP2 can be increased and oxidative stress can be reduced through the activation of AMPK [22]. In addition, AMPK and UCP2 proteins have been shown to have a functionally related mechanism. AMPK is a cellular energy sensor that, when activated, can phosphorylate acetyl-CoA carboxylase and mTOR, downregulate fatty acid synthesis and inhibit the inflammatory cascade [23]; UCP2 dissipates the mitochondrial proton gradient and limits excessive ROS production, and is an important barrier to maintain oxidation–reduction homeostasis [24]. At the same time, AMPK can promote mitochondrial enzyme activity, increase cellular oxidative phosphorylation efficiency, increase UCP2 expression, reduce the body's oxidative stress response, and promote the balance of energy metabolism [25]. In this study, simvastatin upregulated AMPK by 55%, UCP2 by 29%, and significantly reduced MDA and inflammatory cytokines, suggesting that it indirectly weakened platelet activation and excessive coagulation factor activation by restoring mitochondrial function and energy metabolism. The environment reveals the protective effect of simvastatin on the trinity of "metabolization–inflammation–coagulation" in sepsis.

First, this study is a mouse model of sepsis and is difficult to fully extrapolate to a heterogeneous population of sepsis patients; secondly, NF-κB, PI3K/AKT, SIRT1/3 and other pathways have not been detected simultaneously, and the synergistic contribution of such signals to experimental results cannot be ruled out. In the future, it is planned to use the AMPK gene knockout model combined with PI3K inhibitors to further explain cross-regulation of multiple pathways and verify the new targets discovered in this paper in clinical samples.

In summary, simvastatin pretreatment is beneficial to improving platelet activation and hypercoagulation in septic mice.

## Data Availability

No datasets were generated or analysed during the current study.
